# 1279. Real World Treatment Experience of Treatment-Naïve People with HIV who Initiated Treatment with Single Tablet Dolutegravir/Lamivudine in a Test and Treat setting in the US.

**DOI:** 10.1093/ofid/ofac492.1110

**Published:** 2022-12-15

**Authors:** Jennifer Kuretski, Cindy Donovan, Gavin Harper, Deanna Merrill, Katie L Mycock, Alan Oglesby, Aimee Metzner, Jimena Patarroyo

**Affiliations:** Midway Specialty Care Center, West Palm Beach, Florida; ViiV Healthcare, Research Triangle Park, North Carolina; Adelphi Real World, Bollington, England, United Kingdom; ViiV Healthcare, Research Triangle Park, North Carolina; Adelphi Real World, Bollington, England, United Kingdom; ViiV Healthcare, Research Triangle Park, North Carolina; ViiV Healthcare, Research Triangle Park, North Carolina; ViiV Healthcare, Research Triangle Park, North Carolina

## Abstract

**Background:**

Dolutegravir/lamivudine (DTG/3TC) is indicated as a 2DR for both treatment-naïve and virally suppressed PLWH. The feasibility of DTG/3TC use in a Test & Treat (T&T) setting has been demonstrated in a clinical trial, but there is limited evidence with this approach in US real world clinical settings.

**Methods:**

TANDEM was a retrospective medical chart review conducted across 24 US sites. Eligible PLWH were adults initiated on DTG/3TC or DTG/rilpivirine (DTG/RPV) prior to Sept/30/2020 with a minimum clinical follow-up of six months. Treatment-naïve (TN) PLWH had no prior history of HIV therapy. Clinical characteristics, treatment history and outcomes were abstracted. Analyses were descriptive. Reported here are results for the sub-group of TN PLWH that were initiated DTG/3TC as part of a T&T strategy, defined as clinician attestation of treatment initiation shortly after diagnosis and in the absence of known lab values for HIV-1 RNA viral load, CD4 cell count and HIV-1 resistance mutations.

**Results:**

From an overall sample of 469 PLWH on DTG-based 2DR, 318 received DTG/3TC, of whom, 126 were TN and 192 were stable switch. Almost half 48% of PLWH received DTG/3TC as part of a T&T paradigm. Characteristics of the cohort are described in Table 1. In the T&T sub-group, the most common reasons for initiating DTG/3TC were avoidance of long-term toxicities (n=26), followed by simplification/streamlining (n=8) and convenience (n=7). Overall, 114 (90.5%) of TN PLWH achieved the desired health outcome per clinician attestation. At data cut-off, 61 (94%) non T&T achieved virologic suppression, 57 (93%) of the T&T sub-group achieved virological suppression, 3 (5%) did not, and 1 (2%) was still unknown. Of the 3 who did not achieve suppression, 2 remained on DTG/3TC while 1 was switched to bictegravir/emtricitabine/tenofovir alafenamide. Virologic rebound occurred in 6 TN PLWH overall with only 1 occurring in the T&T sub-group.

Baseline Characteristics and Virologic Outcomes

**Figure:**
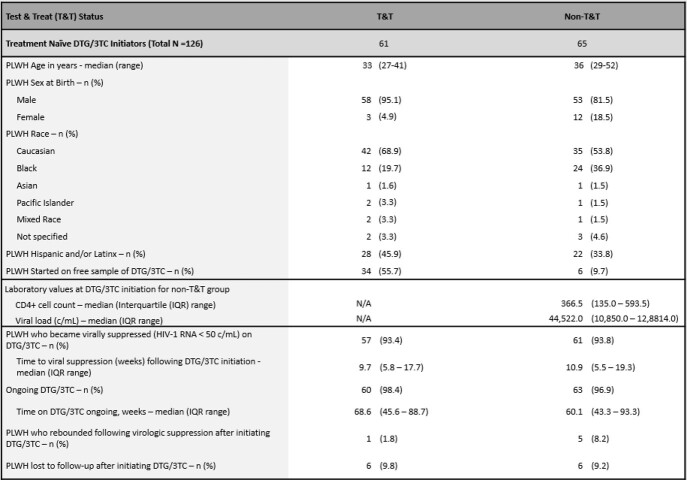

**Conclusion:**

Reflecting results from clinical trials, DTG/3TC achieved its desired health outcomes in the majority of cases regardless of treatment paradigm, with virtually no difference in virological suppression rates across the two cohorts (93-94% achieving suppression in a median duration of 10-11 weeks).

**Disclosures:**

**Cindy Donovan, PharmD**, Johnson & Johnson: Stocks/Bonds|ViiV Healthcare: Employee/Salary|ViiV Healthcare: Stocks/Bonds **Gavin Harper, BA**, ViiV Healthcare: Adelphi Real World were paid consultants (CRO) to conduct the observational research study on behalf of ViiV Healthcare.|ViiV Healthcare: Adelphi Real World were paid consultants (CRO) to conduct the observational research study on behalf of ViiV Healthcare **Deanna Merrill, PharmD, MBA, AAHIVP**, ViiV Healthcare: Salaried employee|ViiV Healthcare: Stocks/Bonds **Katie L. Mycock, MChem**, ViiV Healthcare: Adelphi Real World were paid consultants (CRO) to conduct the observational research study on behalf of ViiV Healthcare|ViiV Healthcare: Adelphi Real World were paid consultants (CRO) to conduct the observational research study on behalf of ViiV Healthcare **Alan Oglesby, MPH**, GlaxoSmithKline (GSK): Employment|GlaxoSmithKline (GSK): Stocks/Bonds **Aimee Metzner, PharmD, AAHIVP**, ViiV Healthcare: Salaried employee|ViiV Healthcare: Stocks/Bonds **Jimena Patarroyo, PharmD, AAHIVP**, ViiV Healthcare: Salaried employee|ViiV Healthcare: Stocks/Bonds|ViiV Healthcare: Stocks/Bonds.

